# Burnett: Essays

**Published:** 1883-01

**Authors:** 


### BOOK NOTICES, REVIEWS, Etc.

Dr. Burnett's Essays, containing "Ecce Medicus," "Natrum Muriaticum," "Gold," "Causes of Cataract," "Curability of Cataract," "Diseases of the Veins," "Supersalinity of the Blood." Price, $2.50. Bœricke & Tafel: New York and Philadelphia. 1882.

These essays are too well known to need any words of comment from us at this late date. We can only say Messrs. Bœricke & Tafel never conferred a greater favor upon American homœopathists than in the republication of Dr. Burnett's essays. These valuable monographs are now for the first time brought together in one volume and are sold at greatly reduced cost. We can only advise our readers to purchase *quickly* and read *slowly*. The money cannot be put to a better use.

